# Addressing health literacy to reduce inequalities among migrants: which profiles need our attention?

**DOI:** 10.1093/eurpub/ckac131.368

**Published:** 2022-10-25

**Authors:** MJ Marques, A Gama, C Cheng, R Osborne, S Dias

**Affiliations:** 1 NOVA National School of Public Health, PHRC, Universidade NOVA de Lisboa, Lisbon, Portugal; 2 Comprehensive Health Research Center, Lisbon, Portugal; 3 Centre of Global Health and Equity, School of Health Sciences, Swinburne University of Technology, Melbourne, Australia

## Abstract

Health literacy, the ability to access, understand, appraise, remember and use health information and services, has great potential to reduce inequalities in access to and outcomes of care. People may have different patterns of health literacy needs and strengths. Yet, the design of interventions is frequently not responsive to the specificities of different segments of the population, including migrant groups. We aimed to identify profiles of migrants to inform the co-design of interventions targeting people at risk of poor outcomes. A cross-sectional survey was conducted with 1126 adult migrants living in Portugal. Data were collected using the 9-dimension HLQ (Health Literacy Questionnaire), and a sociodemographic questionnaire. A cluster analysis of data was performed. Semi-structured interviews were conducted with 15 migrants, stratified by the clusters. The optimal cluster solution yielded 16 profiles revealing diversity in combinations of strengths and needs across the HLQ domains. While 29.8% of migrants scored moderate to high on all 9 domains (profiles 2, 6, 8, 16), 63.4% of participants struggled with 1 or several aspects of health literacy, namely ‘Feeling understood and supported by healthcare providers'. Notably, 36.8% (6 profiles) exhibited difficulty across most of health literacy domains. The interviews provided a tangible description of the health literacy needs and resources with five main themes (access, understand, appraise, retrieve and use). Regarding ‘access', migrants expressed different preferred learning styles and needed to access different sorts of information at distinct times. The ‘use’ of information was rarely a one-time decision but a decision that people needed to make repeatedly. Health literacy is a highly diverse concept where subgroups exhibited diverse patterns. The cluster analysis can be used to inform the co-design, prioritisation and implementation of locally designed, fit-for-purpose solutions to improve health literacy.

**Key messages:**

• Health literacy profiles can inform interventions to mitigate health inequalities among vulnerable migrant groups.

• The identification of distinct profiles can contribute to minimise the disconnect between what people/communities need and what is developed to improve health and equity.

